# Solubility of Cinnarizine in (Transcutol + Water) Mixtures: Determination, Hansen Solubility Parameters, Correlation, and Thermodynamics

**DOI:** 10.3390/molecules26227052

**Published:** 2021-11-22

**Authors:** Faiyaz Shakeel, Mohsin Kazi, Fars K. Alanazi, Prawez Alam

**Affiliations:** 1 Department of Pharmaceutics, College of Pharmacy, King Saud University, Riyadh 11451, Saudi Arabia; mkazi@ksu.edu.sa (M.K.); afars@ksu.edu.sa (F.K.A.); 2 Department of Pharmacognosy, College of Pharmacy, Prince Sattam Bin Abdulaziz University, Al-Kharj 11942, Saudi Arabia; prawez_pharma@yahoo.com

**Keywords:** cinnarizine, {Transcutol-P (1) + water (2)} mixtures, correlation, solubility, thermodynamics

## Abstract

Between 293.2 and 313.2 K and at 0.1 MPa, the solubility of the weak base, cinnarizine (CNZ) (3), in various {Transcutol-P (TP) (1) + water (2)} combinations is reported. The Hansen solubility parameters (HSP) of CNZ and various {(TP) (1) + water (2)} mixtures free of CNZ were also predicted using HSPiP software. Five distinct cosolvency-based mathematical models were used to link the experimentally determined solubility data of CNZ. The solubility of CNZ in mole fraction was increased with elevated temperature and TP mass fraction in {(TP) (1) + water (2)} combinations. The maximum solubility of CNZ in mole fraction was achieved in neat TP (5.83 × 10^−2^ at 313.2 K) followed by the minimum in neat water (3.91 × 10^−8^ at 293.2 K). The values of mean percent deviation (*MPD*) were estimated as 2.27%, 5.15%, 27.76%, 1.24% and 1.52% for the “Apelblat, van’t Hoff, Yalkowsky–Roseman, Jouyban–Acree, and Jouyban–Acree–van’t Hoff models”, respectively, indicating good correlations. The HSP value of CNZ was closed with that of neat TP, suggesting the maximum solubilization of CNZ in TP compared with neat water and other aqueous mixtures of TP and water. The outcomes of the apparent thermodynamic analysis revealed that CNZ dissolution was endothermic and entropy-driven in all of the {(TP) (1) + water (2)} systems investigated. For {(TP) (1) + water (2)} mixtures, the enthalpy-driven mechanism was determined to be the driven mechanism for CNZ solvation. TP has great potential for solubilizing the weak base, CNZ, in water, as demonstrated by these results.

## 1. Introduction

**Figure 1:**
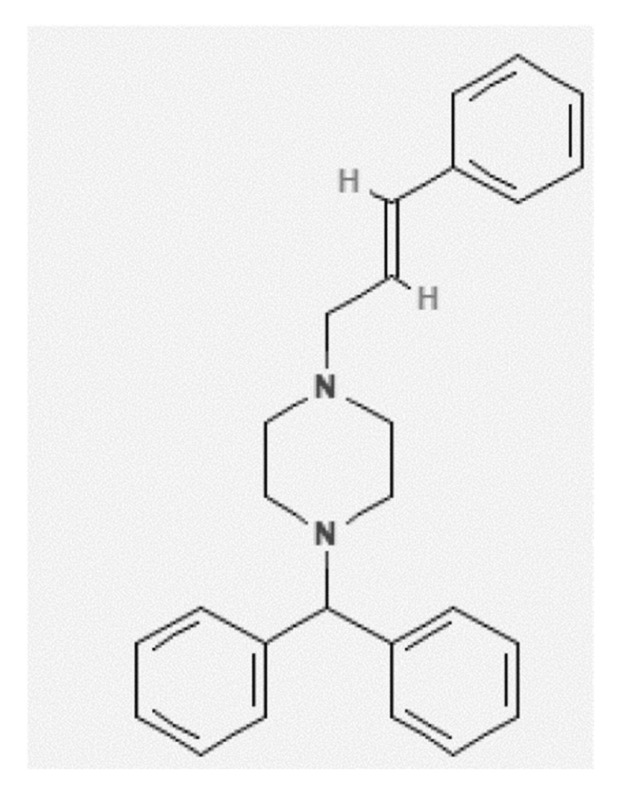
Molecular structure of cinnarizine (CNZ).

Cinnarizine (CNZ) (Figure 1, IUPAC name: 1-benzhydryl-4-[(*E*)-3-phenylprop-2-enyl]piperazine, CAS number: 298-57-7, PubChem CID: 1547484, molecular formula: C_26_H_28_N_2_, and molar mass: 368.50 g mol^−1^) appears as a white crystalline powder [1,2]. It is used as an antihistaminic and blood-flow promoter [2,3]. The biopharmaceutical classification system (BCS) classifies it as a BCS class II drug, meaning it has poor aqueous solubility and high permeability [1,4]. It is a weak base, which is practically insoluble in water with a high partition coefficient value (log P = 5.8) [5]. Hence, the bioavailability and oral absorption of CNZ are limited by its low solubility and poor dissolution rate [1]. CNZ is a non-efficient drug molecule due to its low solubility, stability, and poor bioavailability from a physicochemical viewpoint [1,2,3,4,5]. Various lipid-based drug delivery systems, such as self-nanoemulsifying drug delivery systems (SNEDDS) and solidified SNEDDS, have been developed to modify the physicochemical characteristics of CNZ [2,6,7,8,9,10,11,12,13,14].

Physicochemically, the solubility of active pharmaceutical compounds is an important property for the purification, production, dosage form design, and application of these compounds [1,15,16]. The literature on the solubility data of CNZ in aqueous cosolvent mixtures is limited. It has pH-dependent solubility in aqueous media [2]. Its solubility increases with decreases in pH, and vice versa. The solubility of CNZ has been reported as 0.29 mg mL^−1^ in an aqueous buffer with pH = 2.0, 0.017 mg mL^−1^, pH = 5.0, and 0.002 mg mL^−1^, pH = 6.5 at 310.2 K [2]. The solubility of CNZ has also been reported in water and four organic solvents such as acetonitrile, butyl acetate, 1-butanol, and 2-propanol between 288.15 and 313.15 K [1].

The solubility values and thermodynamic properties of CNZ (3) are not known in various {Transcutol-P (TP) (1) + water (2)} mixtures from 293.2–313.2 K and at 0.1 MPa. Thus, this study evaluated the equilibrium solubility values and thermophysical characteristics of CNZ (3) in various {TP (1) + water (2)} combinations along with pure TP and pure water between 293.2 and 313.2 K and at 0.1 MPa. From a pharmaceutical point of view, TP is a safe and Food and Drug Administration (FDA)-approved solubilizer [2]. It is used as a potential solubilizer/cosolvent in the preparation of various lipid-based drug delivery systems [2,9,17]. Recently, it has also been studied as a potential solubilizer in the solubility enhancement of various poorly soluble drugs, including sunitinib malate, flufenamic acid, sinapic acid, apremilast, ketokonazole, and sulphadiazine [18,19,20,21,22,23]. Due to these reasons, it was selected as a cosolvent in this study.

## 2. Results and Discussion

### 2.1. Mole Fraction Solubility Data of CNZ

Between 293.2 and 313.2 K and atmospheric pressure, Table 1 lists the solubility values of CNZ in mole fraction (3) in binary {TP (1) + water (2)} combinations, including neat TP and neat water. The solubility of CNZ in mole fraction (3) in different {TP (1) + water (2)} combinations at various temperatures is unknown. However, the solubility of CNZ in mole fractions in water has been measured at various temperatures [1]. The mole fraction solubility of CNZ in water was reported to be 6.63 × 10^−8^, 7.71 × 10^−8^, and 9.35 × 10^−8^ at 298.3 K, 303.0 K, and 307.8 K, respectively [1]. The mole fraction solubility of CNZ in water was determined to be 5.67 × 10^−8^, 7.82 × 10^−8^, and 9.78 × 10^−8^ at three closed temperatures of 298.2 K, 303.2 K, and 308.2 K, respectively. In neat water, these CNZ mole fraction solubility values were similar to those previously reported in the literature [1].

**Figure 2:**
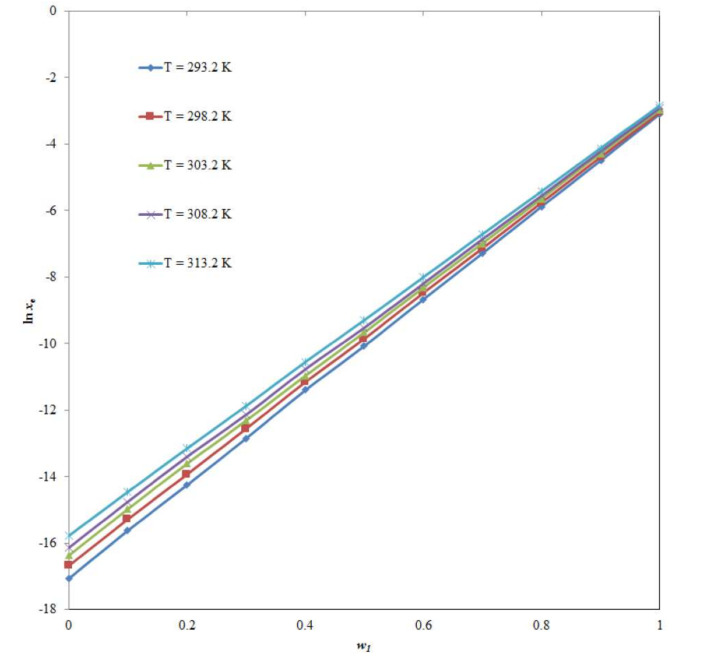
Influence of Transcutol-P (TP) mass fraction (*w*_1_) on logarithmic solubilities of CNZ between 293.2 and 313.2 K.

The mole fraction solubility of CNZ was determined to be the lowest in neat water and the highest in neat TP. The low polarity of TP relative to the high polarity of water may explain the maximal CNZ solubility in neat TP [18,19]. The solubility of CNZ (3) in binary {TP (1) + water (2)} combinations was observed to increase with elevated temperatures and increase in TP mass fraction at constant pressure (0.1 MPa) between 293.2 and 313.2 K. Between 293.2 and 313.2 K, the effect of TP mass fraction on the logarithmic mole fraction solubility of CNZ was also investigated, and the findings are shown in Figure 2. At all five temperatures tested, the logarithmic solubility of CNZ in mole fraction was enhanced consistently with an increase in TP mass fraction in binary {TP (1) + water (2)} combinations. The logarithmic solubility of CNZ in mole fraction was likewise shown to enhance significantly from pure water to pure TP. As a result, TP has the potential to be employed as a solubilizer/cosolvent in the solubilization of CNZ in water.

### 2.2. Hansen Solubility Parameters (HSPs)

The total HSP (*δ*_t_) for CNZ was estimated to be 19.40 MPa^1/2^ using HSPiP software and Equation (1). HSP values for neat TP (*δ*_1_) and neat water (*δ*_2_) were anticipated to be 21.40 and 47.80 MPa^1/2^, respectively. Equation (2) was used to calculate the HSP value for various {TP (1) + water (2)} combinations free of CNZ (*δ*_mix_). The *δ*_mix_ values were estimated to be between 24.04 and 45.16 MPa^1/2^. Overall, the HSP of neat TP (*δ*_1_ = 21.40 MPa^1/2^) and CNZ (*δ*_t_ = 19.40 MPa^1/2^) were very close. The solubility of CNZ in neat TP was likewise found to be the highest in the experiments. As a result, these findings were in good accord with the CNZ solubility data obtained from experiments with {TP (1) + water (2)} combinations.

### 2.3. Cosolvency-Based Mathematical Models for CNZ Solubility Correlation

Five distinct cosolvency-based mathematical models, including the modified “Apelblat, van’t Hoff, Yalkowsky–Roseman, Jouyban–Acree, and Jouyban–Acree–van’t Hoff models” were used to link the measured solubility values of CNZ [18,19,20,24,25,26,27]. Table 2 summarizes the results for the correlation of CNZ in binary {TP (1) + water (2)} combinations with the modified “Apelblat model”. The overall mean percent deviation (*MPD*) for this model was estimated to be 2.27%. The determination coefficient (*R*^2^) for CNZ (3) in all cosolvent combinations with neat solvents was obtained at between 0.9955 and 0.9998. These findings revealed a strong connection between the experimental CNZ (3) solubility data and the modified “Apelblat model” in binary {TP (1) + water (2)} combinations.

Table 3 summarizes the results for the correlation of CNZ in binary {TP (1) + water (2)} combinations with the “van’t Hoff model”. The overall *MPD* for the “van’t Hoff model” was estimated to be 5.15%. The *R*^2^ for CNZ (3) in all cosolvent mixtures with neat solvents was obtained at between 0.9947 and 0.9993. These findings also revealed a strong connection between experimental CNZ (3) solubility data and the “van’t Hoff model” in binary {TP (1) + water (2)} combinations.

Table 4 summarizes the results for the correlation of CNZ in binary {TP (1) + water (2)} combinations with the “Yalkowsky–Roseman model”. The *MPD* for this model was estimated to be 24.76%, also showing a strong connection between experimental CNZ (3) solubility data and the “Yalkowsky–Roseman model” in binary {TP (1) + water (2)} combinations.

The solubility values of CNZ (3) in {TP (1) + water (2)} compositions at various temperatures and cosolvent compositions can also be correlated using the “Jouyban–Acree and Jouyban–Acree–van’t Hoff models” [28]. The overall *MPDs* were determined as 1.24% and 1.52% for “Jouyban–Acree and Jouyban–Acree–van’t Hoff models”, respectively. The overall *MPD* for the “Yalkowsky–Roseman model” was the highest compared with the other models studied. In the “Yalkowsky–Roseman model”, the model parameters were not utilized (equal to zero) compared to the other models studied. Therefore, the highest *MPD* value for the “Yalkowsky–Roseman model” was due to the fact that this model did not utilize any model parameters for the calculation of *MPD* [19,26].

### 2.4. Apparent Thermodynamic Parameters for CNZ

**Figure 3:**
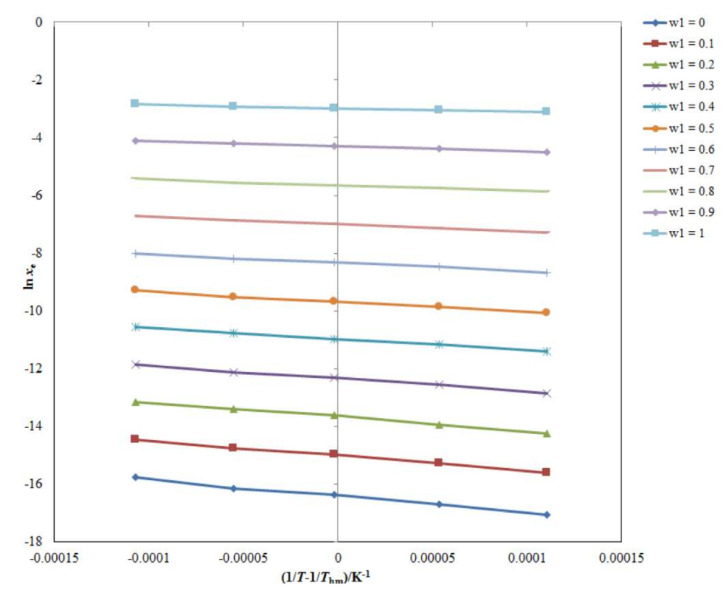
Van’t Hoff curves for logarithmic solubility of CNZ (3) in aqueous mixtures of TP (1) and water (2).

The apparent standard enthalpy (Δ_soln_*H*°) values for CNZ (3) in all cosolvent mixtures, including neat solvents, were calculated using the van’t Hoff technique. As reported in Table 5, Figure 3 displays the linear van’t Hoff curves of CNZ (3) in all cosolvent compositions and pure solvents where R^2^ was more than 0.990. Table 5 also includes the values of all thermodynamic quantities. The CNZ (3) Δ_soln_*H*° values in binary {TP (1) + water (2)} combinations with pure solvents ranged from 9.719 to 47.65 kJ mol^−1^. In various {TP (1) + water (2)} mixtures including neat solvents, the apparent standard Gibbs energy (Δ_soln_*G*°) values for CNZ (3) were computed between 7.492 and 41.32 kJ mol^−1^. The endothermic dissolution of CNZ (3) in various {TP (1) + water (2)} combinations with pure solvents was demonstrated by the obtained values of Δ_soln_*H*° for CNZ [18,19]. The Δ_soln_*H*° and Δ_soln_*G*° values are inversely proportional to the solubility of the solute. Hence, the maximum Δ_soln_*H*° and Δ_soln_*G*° values for CNZ were obtained in neat water compared to the neat TP. The apparent standard entropy (Δ_soln_*S*°) values for CNZ (3) in binary {TP (1) + water (2)} combinations with neat solvents were computed between 7.346 and 23.94 J mol^−1^ K^−1^, implying the entropy-driven dissolution of CNZ (3) in diverse {TP (1) + water (2)} combinations with pure solvents [18]. Finally, in all {TP (1) + water (2)} combinations, including pure solvents, the dissolution of CNZ (3) was reported to be endothermic and entropy-driven [18,19].

### 2.5. Enthalpy–Entropy Compensation Analysis

**Figure 4:**
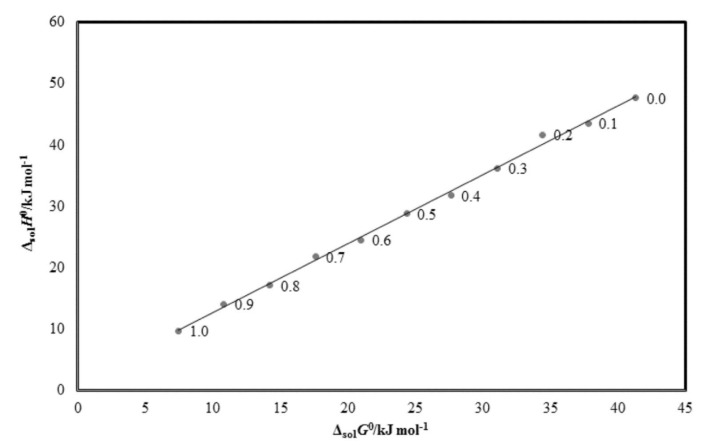
Δ_sol_*H*° vs. Δ_sol_*G*° enthalpy–entropy compensation plot for solubility of CNZ in various {TP (1) + water (2)} mixtures at *T*_hm_ = 303.0 K.

An enthalpy–entropy compensation study was used to analyze the solvation behavior/cosolvent action of CNZ (3) in binary {TP (1) + water (2)} combinations with pure solvents, and the findings are shown in Figure 4. Figure 4 shows that CNZ (3) offers a linear Δ_soln_*H*° vs. Δ_soln_*G*° trend in all {TP (1) + water (2)} combinations with pure solvents, with a slope value of 1.124 and *R*^2^ = 0.997. Based on these findings, the driving mechanism for CNZ (3) solvation in all {TP (1) + water (2)} combinations, including neat solvents, is assumed to be enthalpy-driven. This method of CNZ solvation could be explained by the fact that CNZ solvates best in neat TP molecules compared to neat water molecules [19,28]. As a result, the molecular interactions between CNZ–TP molecules were stronger than those between CNZ–water molecules. This solvation behavior of CNZ (3) in binary {TP (1) + water (2)} combinations with pure solvents was identical to that of flufenamic acid, piperine, sinapic acid, sunitinib malate, apigenin, and apremilast in binary {TP (1) + water (2)} combinations [18,19,20,29,30,31].

## 3. Materials and Methods

### 3.1. Materials

CNZ (mass fraction purity > 0.99 by HPLC) was procured from FDC Ltd. (Mumbai, India). TP (mass fraction purity > 0.99 by GC) was obtained from Gattefosse (Lyon, France). The water utilized in this research was deionized and came from the laboratory’s Milli-Q unit. Table 6 summarizes the materials information.

### 3.2. CNZ (3) Solubility Determination in Binary {TP (1) + Water (2)} Combinations

Using a Digital Analytical Balance (Mettler Toledo, Greifensee, Switzerland) with a sensitivity of 0.10 mg, all {TP (1) + water (2)} combinations were created on a mass basis. The mass fraction of TP used to make various {TP (1) + water (2)} compositions ranged from 0.10–0.90. Three replicates of each {TP (1) + water (2)} composition were made.

Using a standard shake-flask method [32], the mole fraction solubility of CNZ against the mass fraction of TP (*w*_1_ = 0.0–1.0; *w*_1_ is TP mass fraction in {TP (1) + water (2)} compositions) and pure solvents was tested from 293.2–313.2 K and at 0.1 MPa in various {TP (1) + water (2)} mixtures and pure solvents. Extra CNZ crystals were mixed with known amounts of each {TP (1) + water (2)} composition and neat solvents. Three repetitions of each experiment were carried out. Inside the Biological Shaker (Julabo, PA, USA), the acquired samples were saturated for three days to achieve equilibrium. After reaching equilibrium, the saturated samples were withdrawn from the shaker and centrifuged at 5000 rpm. The supernatants were withdrawn, diluted (wherever applicable), and used for the estimation of CNZ content using a reported HPLC method at 253 nm [12]. The mole fraction solubilities (*x*_e_) of CNZ were calculated using their standard formulae [20,33].

### 3.3. HSPs of CNZ and Various {TP (1) + Water (2)} Mixtures

The HSP of a pharmaceutical compound is associated with its solubility in neat solvent or aqueous-cosolventmixtures. It is well-known that the closed value of the HSP of a pharmaceutical compound with that of a particular solvent could result in the maximum solubility of a pharmaceutical compound in that particular solvent [34]. Hence, the HSP for CNZ, neat TP, and neat water were predicted in this research. The *δ*_t_ value for CNZ, neat TP, and neat water was predicted using Equation (1) [35,36,37,38]:

(1) $$\delta_{t}^{2}=\delta_{d}^{2}+\delta_{p}^{2}+\delta_{h}^{2}$$

where *δ*_d_ = dispersion HSP; *δ*_p_ = polar HSP, and *δ*_h_ = hydrogen-bonded HSP. These values for CNZ and neat solvents were predicted utilizing HSPiP software (version 4.1.07, Louisville, KY, USA) by entering the simplified molecular input line entry system (SMILES) of each component into the HSPiP system [36].

The HSP for various {TP (1) + water (2)} mixture free of CNZ (*δ*_mix_) was calculated using Equation (2) [38]:

(2) $$\delta_{\mathrm{mix}}=\propto \delta_{1}+\left(1-\propto \right)\delta_{2}$$

where, *α* = volume fraction of TP in {TP (1) + water (2)} mixture; *δ*_1_ = HSP of neat TP, and *δ*_2_ = HSP of neat water.

### 3.4. Cosolvency-Based Mathematical Models for CNZ Solubility Correlation

The mathematical correlation of experimental data of pharmaceutical compounds is important for practical predictions/validations [20,33,39,40]. As a result, the measured solubility values of CNZ were predicted using the modified “Apelblat, van’t Hoff, Yalkowsky–Roseman, Jouyban–Acree, and Jouyban–Acree–van’t Hoff models” [18,19,20,24,25,26,27].

The “Apelblat model solubility (*x*^Apl^)” of CNZ (3) in binary {TP (1) + water (2)} combinations was predicted using Equation (3) [24,25]:

(3) $$\ln\;x^{\mathrm{Apl}}=A+\frac{B}{T}+C\ln\left(T\right)$$

where *A*, *B,* and *C* are the model parameters of Equation (3), which were determined using nonlinear multivariate regression analysis of experimental solubility data of CNZ summarized in Table 1 [18]. The correlation between *x*_e_ and *x*^Apl^ of CNZ was performed using *MPD*. The *MPD* was calculated using its reported formula [27].

The “van’t Hoff model solubility (*x*^van’t^)” of CNZ (3) in binary {TP (1) + water (2)} combinations is predicted using Equation (4) [20]:

(4) $$\ln x^{\mathrm{van}’t}=a+\frac{b}{T}$$

where *a* and *b* are the Equation (4) parameters, which were found using the least square technique [19]. The solubility values in a specific solvent combination at different temperatures are represented by Equations (3) and (4), and there is no way to forecast the solubility values in other solvent mixtures of binary solvent composition.

The logarithmic solubility of the “Yalkowsky–Roseman model (log *x*^Yal^)” for CNZ (3) in various {TP (1) + water (2)} combinations was predicted by Equation (5) [26]:

(5) $$\log x^{\mathrm{Yal}}=w_{1}\log x_{1}+w_{2}\log x_{2}$$

where *x*_1_ = the solubility of CNZ (3) in TP (1); *x*_2_ = the solubility of CNZ in water (2); *w*_1_ = TP mass fraction, and *w*_2_ = water mass fraction. Equation (5) models the solubility values of pharmaceutical compounds in different solvent mixtures at a given temperature.

The “Jouyban–Acree model” correlates the solubility of pharmaceutical compounds at the solvent compositions as well as temperature (*x*_m,T_), and was predicted using Equation (6) [27]:

(6) $$\ln x_{m,T}=w_{1}\ln x_{1,T}+w_{2}\ln x_{2,T}+\left(\frac{w_{1}.w_{2}}{T}\right)\sum_{i=0}^{2}J_{i}{\left(w_{1}-w_{2}\right)}^{i}$$

where *x*_1,T_ and *x*_2,T_ are the solubility of CNZ in TP (1) and water (2) at temperature *T*, and the symbols *J* are the model parameters. The solubility values of CNZ in pure solvents are required as input data to predict the solubility of CNZ in cosolvent compositions at the temperature of interest. To overcome this constraint, Equations (2) and (6) can be combined to form the “Jouyban–Acree–van’t Hoff model” [27].

### 3.5. Apparent Thermodynamic Parameters for CNZ

At the mean harmonic temperature (*T*_hm_), all apparent thermodynamic parameters were examined. The *T*_hm_ was calculated using the usual formula [27]. In this study, the *T*_hm_ was found to be 303.0 K. An apparent thermodynamic analysis was used to calculate several apparent thermodynamic parameters. The van’t Hoff and Gibbs equations were used to conduct this analysis. Equation (7) was used to determine the Δ_soln_*H*° values for CNZ (3) in binary {TP (1) + water (2)} combinations at *T*_hm_ = 303.0 K using the van’t Hoff methodology [28,41]:

(7) $${\left(\frac{\partial \ln x_{e}}{\partial \left(\frac{1}{T}-\frac{1}{T_{\mathrm{hm}}}\right)}\right)}_{P}=-\frac{\Delta_{\mathrm{soln}}H{}^{\circ}}{R}$$

By plotting ln *x*_e_ values of CNZ vs. (1/*T*−1/*T*_hm_), the Δ_soln_*H*° and Δ_soln_*G*° values for CNZ were calculated from the slope and intercept, using the following Equations (8) and (9), respectively [28,41]:

(8) $$\Delta_{\mathrm{soln}}H{}^{\circ}=-R{\left(\frac{\partial \ln x_{e}}{\partial \left(\frac{1}{T}-\frac{1}{T_{\mathrm{hm}}}\right)}\right)}_{P}$$

(9) $$\Delta_{\mathrm{soln}}G{}^{\circ}=-RT_{\mathrm{hm}}\cdot \mathrm{intercept}$$

Equation (10) was used to calculate the Δ_soln_*S*° values for CNZ (3) in binary {TP (1) + water (2)} combinations [28,41,42]:

(10) $$\Delta_{\mathrm{soln}}S{}^{\circ}=\frac{\Delta_{\mathrm{soln}}H{}^{\circ}-\Delta_{\mathrm{soln}}G{}^{\circ}}{T_{\mathrm{hm}}}$$

### 3.6. Enthalpy–Entropy Compensation Analysis

An enthalpy–entropy compensation analysis was used to analyze the solvation behavior of CNZ (3) in binary {TP (1) + water (2)} combinations, as previously proposed [16]. This analysis was carried out by plotting the weighted graphs of Δ_soln_*H*° vs. Δ_soln_*G*° at *T*_hm_ = 303.0 K [17,26].

## 4. Conclusions

In the literature, there is scarce data concerning the solubility of CNZ in diverse aqueous cosolvent mixtures. As a result, the mole fraction solubility data of a weak base, CNZ, (3) in binary {TP (1) + water (2)} combinations including pure solvents was determined in this investigation from 293.2–313.2 K and at 0.1 MPa. In all {TP (1) + water (2)} compositions, including pure solvents, the mole fraction solubilities of CNZ (3) increased with the rise in temperature and TP mass fraction. At each temperature tested, the maximum and minimum mole fraction solubility of CNZ were found in neat TP and neat water, respectively. In all {TP (1) + water (2) combinations including pure solvents, experimentally determined CNZ (3) solubility data correlated well with the “Apelblat, van’t Hoff, Yalkowsky–Roseman, Jouyban–Acree, and Jouyban–Acree–van’t Hoff models”. In all {TP (1) + water (2)} combinations, including pure solvents, the dissolution behavior of CNZ was endothermic and entropy-driven. In all {TP (1) + water (2)} combinations, including pure solvents, the predominant mechanism for CNZ solvation capacity was enthalpy-driven.

## Figures and Tables

**Table 1:** Solubility values (*x*_e_) of cinnarizine (CNZ) in mole fraction (3) in binary {Transcutol-P (TP) (1) + water (2)} combinations from 293.2–313.2 K and at 0.1 MPa ^a^.

*w*_1_^a^	*x*_e_^b^				
	*T* = 293.2 K	*T* = 298.2 K	*T* = 303.2 K	*T* = 308.2 K	*T* = 313.2 K
0.0	3.91 × 10^−8^	5.67 × 10^−8^	7.82 × 10^−8^	9.78 × 10^−8^	1.42 × 10^−7^
0.1	1.66 × 10^−7^	2.30 × 10^−7^	3.16 × 10^−7^	3.91 × 10^−7^	5.30 × 10^−7^
0.2	6.50 × 10^−7^	8.87 × 10^−7^	1.23 × 10^−6^	1.51 × 10^−6^	1.95 × 10^−6^
0.3	2.64 × 10^−6^	3.50 × 10^−6^	4.49 × 10^−6^	5.35 × 10^−6^	7.00 × 10^−6^
0.4	1.12 × 10^−5^	1.42 × 10^−5^	1.72 × 10^−5^	2.09 × 10^−5^	2.62 × 10^−5^
0.5	4.24 × 10^−5^	5.26 × 10^−5^	6.34 × 10^−5^	7.33 × 10^−5^	9.22 × 10^−5^
0.6	1.73 × 10^−4^	2.10 × 10^−4^	2.44 × 10^−4^	2.77 × 10^−4^	3.36 × 10^−4^
0.7	6.89 × 10^−4^	8.00 × 10^−4^	9.22 × 10^−4^	1.05 × 10^−3^	1.23 × 10^−3^
0.8	2.81 × 10^−3^	3.17 × 10^−3^	3.53 × 10^−3^	3.90 × 10^−3^	4.43 × 10^−3^
0.9	1.12 × 10^−2^	1.25 × 10^−2^	1.38 × 10^−2^	1.48 × 10^−2^	1.62 × 10^−2^
1.0	4.52 × 10^−2^	4.78 × 10^−2^	5.08 × 10^−2^	5.44 × 10^−2^	5.83 × 10^−2^

^a^ The uncertainties *u* are *u*(*T*) = 0.2 K, *u*(*w*_1_) = 0.0007, and *u*(*p*) = 2 kPa. ^b^ The relative uncertainty *u*_r_ in solubility is *u*_r_(*x*_e_) = 0.016.

**Table 2:** Results for the modified “Apelblat model” for CNZ (3) in various {TP (1) + water (2)} combinations.

w_1_	A	B	C	*R* ^2^	MPD (%)
0.0	224.09	−15741	−32.997	0.9956	-
0.1	286.07	−18057	−42.264	0.9979	-
0.2	442.94	−24887	−65.536	0.9980	-
0.3	211.39	−13815	−31.176	0.9969	-
0.4	−25.275	−2611.0	4.0112	0.9988	-
0.5	8.3136	−3761.7	−0.97648	0.9957	2.27
0.6	53.365	−5294.9	−7.7389	0.9956	-
0.7	−72.315	715.43	11.018	0.9955	-
0.8	−46.705	101.83	7.1263	0.9981	-
0.9	127.04	−7373.2	−18.728	0.9994	-
1.0	−132.56	4855.2	19.875	0.9998	-

**Table 3:** Resulting data for “van’t Hoff model” for CNZ (3) in different {TP (1) + water (2)} combinations.

w_1_	a	b	*R* ^2^	MPD (%)
0.0	2.5154	−5733.0	0.9947	
0.1	2.2793	−5240.5	0.9967	
0.2	2.8838	−5015.9	0.9953	
0.3	2.0440	−4360.6	0.9959	
0.4	1.6487	−3824.2	0.9987	
0.5	1.7473	−3462.8	0.9954	5.15
0.6	1.3939	−2946.3	0.9952	
0.7	1.6646	−2623.1	0.9993	
0.8	1.1397	−2057.0	0.9978	
0.9	1.2918	−1694.4	0.9973	
1.0	0.88450	−1169.3	0.9960	

**Table 4:** Resulting data for “Yalkowsky–Roseman model” for CNZ (3) in different {TP (1) + water (2)} combinations from 293.2–313.2 K.

*w*_1_	log *x*^Yal^					MPD (%)
	*T* = 293.2 K	*T* = 298.2 K	*T* = 303.2 K	*T* = 308.2 K	*T* = 313.2 K	*-*
0.1	−6.80	−6.65	−6.52	−6.43	−6.28	-
0.2	−6.19	−6.06	−5.94	−5.86	−5.72	-
0.3	−5.58	−5.46	−5.36	−5.28	−5.16	-
0.4	−4.98	−4.87	−4.78	−4.71	−4.60	24.76
0.5	−4.37	−4.28	−4.20	−4.13	−4.04	-
0.6	−3.77	−3.69	−3.61	−3.56	−3.47	-
0.7	−3.16	−3.09	−3.03	−2.98	−2.91	-
0.8	−2.55	−2.50	−2.45	−2.41	−2.35	-
0.9	−1.95	−1.91	−1.87	−1.83	−1.79	-

**Table 5:** Apparent standard enthalpy (Δ_soln_*H*°), apparent standard Gibbs energy (Δ_soln_*G*°), apparent standard entropy (Δ_soln_*S*°), and van’t Hoff *R*^2^ values for CNZ (3) in different {TP (1) + water (2)} combinations at *T*_hm_ = 303.0 K ^a^.

w_1_	Δ_soln_*H*°/kJ mol^−1^	Δ_soln_*G*°/kJ mol^−1^	Δ_soln_*S*°/J mol^−1^ K^−1^	*R* ^2^
0.0	47.65	41.32	20.48	0.994
0.1	43.56	37.82	18.92	0.996
0.2	41.69	34.43	23.94	0.995
0.3	36.24	31.10	16.96	0.995
0.4	31.78	27.64	13.68	0.998
0.5	28.78	24.38	14.50	0.995
0.6	24.49	20.98	11.57	0.995
0.7	21.80	17.61	13.82	0.999
0.8	17.09	14.23	9.464	0.997
0.9	14.08	10.83	10.72	0.997
1.0	9.719	7.492	7.346	0.996

^a^ The relative uncertainties are *u*_r_(Δ_soln_*H*^0^) = 0.043, *u*_r_(Δ_soln_*G*^0^) = 0.045, and *u*_r_(Δ_soln_*S*^0^) = 0.034.

**Table 6:** Materials list.

Material	Molecular Formula	Molar Mass (g mol^−1^)	CAS RN	Purification Method	Mass Fraction Purity	Analysis Method	Source
CNZ	C_26_H_28_N_2_	368.50	298-57-7	None	>0.99	HPLC	FDC Ltd.
TP	C_6_H_14_O_3_	134.17	111-90-0	None	>0.99	GC	Gattefosse
Water	H_2_O	18.07	7732-18-5	None	-	-	Milli-Q

CNZ: cinnarizine; TP: Transcutol-P; HPLC: high-performance liquid chromatography; GC: gas chromatography.

## Data Availability

This study did not report any data.

## Sample Availability

Samples of the compounds CNZ are available from the authors.
